# The neuroscience of algorithmic suffering: short comparative analysis between human and AI

**DOI:** 10.3389/fpsyg.2025.1718823

**Published:** 2025-12-04

**Authors:** Esen K. Tütüncü, Mar Gonzalez-Franco

**Affiliations:** 1 Institute of Neurosciences, Universitat de Barcelona, Barcelona, Spain; 2 Google, Seattle, WA, United States

**Keywords:** neuroscience, artificial intelligence, reinforcement learning, predictive processing, consciousness, suffering, behavioral psychology

## Abstract

Across cultures and centuries, humans have sought to explain suffering, be it as moral failure, biological necessity, or existential condition. Today, as artificial intelligence begins to mimic aspects of thought and emotion, the question resurfaces: can a machine suffer? In this paper, we revisit suffering not as a sentimental analogy but as a comparative lens between human and algorithmic cognition. Building on earlier reflections on “painful intelligence”, we examine how frustration, reward, and prediction take shape in neural and computational systems, grounding our analysis in Bayesian inference, behavioral psychology, and theories of consciousness. While both humans and machines respond to errors and unmet goals, only the former experience these as violations of meaning and integrity. By tracing this divide, we suggest that suffering remains the last frontier between optimization and awareness—a reminder that consciousness cannot be reduced to performance, however convincing the imitation.

## 1 What is human suffering?

We should first establish two distinct definitions of suffering, also presented in Hyvärinen (2022). The first, rooted in Buddhist and Stoic traditions, sees suffering as arising from frustration—a feeling of not getting what you want, desires left unmet (Rāhula, 1974; Aurelius, 2015). The second, associated with contemporary philosopher Eric Cassell, defines suffering as any perceived threat to a person's integrity (Cassell, 1998; Scarry, 2020).

Of the two types of suffering, we focus primarily on the latter, as it shifts the concept from a simple series of unmet goals to a more existential threat to human integrity. This definition extends beyond unfulfilled desires to include emotional well-being, physical health, social roles, and even operational coherence. In AI terms, this would be similar to an agent's ability to achieve its objective. In both humans and machines, an intelligent agent can take action to change its environment. Both are assigned goals, but there are critical differences in how those goals are structured. Humans may aim to become idealized versions of themselves, ascending Maslow's hierarchy toward self-actualization (Maslow, 1943). AI, on the other hand, is typically programmed to optimize a specific task. Just as human plans can go awry, AI systems can also encounter obstacles when their programmed objectives are not met.

Here lies a crucial asymmetry: when an AI network fails to converge, it has no awareness of this failure and thus does not suffer. Instead, the frustration shifts to the human training it, who must reconcile the failed goal. The feeling we associate with being distinctly human (frustration or suffering) requires conscious awareness to be truly experienced. While humans can register failures unconsciously, the full phenomenology of suffering demands that we know we are failing. This distinction is essential: without consciousness, AI cannot truly suffer, even when its processes mimic the conditions that cause suffering in us.

## 2 Rewards

Beyond the view that consciousness is not yet achievable through technology, we can examine foundational overlaps. At the core of AI is a machine learning algorithm that minimizes error. An image classifier reduces false positives or negatives through iterative improvement. Reinforcement learning systems aim to maximize rewards, a structure that mimics human behavior, where we are also reward-driven, though this pursuit often ends in disappointment (Sutton and Barto, 1998). When an AI receives less reward than expected, it experiences a negative Reward Prediction Error (RPE), analogous to a letdown. In humans, dopamine encodes this prediction gap, while in AI, network weights are adjusted accordingly (Ferreri et al., 2019). The reward mechanisms differ: humans rely on neurochemistry, AI on optimization. Yet both reflect the mismatch between expectation and outcome.

Although the RPE model is simple, it highlights a key difference: in humans it is neurochemical, while in AI it is purely numerical. Still, both reveal important insights into suffering. Humans pursue intrinsic rewards that emerge from curiosity, values, and meaning. Newer AI models attempt to simulate internal goals (Singh et al., 2009; Oudeyer et al., 2016), but traditional architectures remain limited to external optimization. In some modern architectures, reinforcement learning from human feedback (RLHF) allows models to align their outputs with human preferences rather than raw optimization, a process that introduces a faint echo of social reward. Both systems can fall into repetitive cycles of goal-seeking that do not produce lasting satisfaction. For AI, this happens during training until convergence. For humans, the process is more complex. We do not always choose the most efficient path, but often seek meaning along the way. Our desires can lead to chronic dissatisfaction, especially when they stem from evolutionary cravings for rewards that no longer serve us. While primary rewards like sugar ensured survival, they can now cause an imbalance. Secondary rewards such as money or power depend on social dynamics, often leaving people feeling powerless. In comparison, AI may appear to crave “numerical sugar,” the transient satisfaction of optimized reward signals, while humans strive for goals shaped by culture and relationships. Misalignment between goals and wellbeing affects both. Some people pursue hollow values and never find fulfillment. Others experience meaning through others, such as shared victories, faith, or empathy, reflecting the social richness of human motivation.

## 3 Risk

It is true that human suffering doesn't have to be active; it can be anticipated, based on human self-projection and simulation of other scenarios, or even counterfactual thinking after grief or traumatic events, which we also call: anxiety. In any context of intelligence, this manifests as risk aversion: the tendency to prefer a safer choice over a gamble, even if the gamble could mean a bigger reward. And that is valid for AI as well. A threat in AI terms is the prediction of future frustration, which is a form of backward propagation. This is the computational version of our own anxious tendencies, the “what ifs.”

Human thinking involves two concurrent processes: a fast, intuitive system that handles snap judgments, and a slower, more deliberate system for reasoning (Kahneman, 2011). The fast system helps us react quickly without analyzing every detail, while the slower one supports logical, reflective decisions. This dual structure loosely resembles AI architectures. Symbolic AI mimics the logical, rule-based mode but struggles with complexity. Neural networks, like our intuitive system, are powerful but depend on large data inputs. The Bayesian brain model suggests that humans, like AI, constantly update beliefs based on new evidence (Friston, 2010). In this view, the brain strives to minimize “free energy,” a measure of surprise or uncertainty in perception and action. However, belief updating in humans is emotionally weighted and often biased. People do not always incorporate negative or disconfirming information, particularly in polarized groups. This resistance reveals why suffering in humans cannot be reduced to error correction: it is shaped by meaning, emotion, and the desire to protect one's worldview.

An AI confined to “slow thinking” alone can never experience the same degree of frustration that arises from the errors of human “fast thinking.” While an AI will still be making serious errors on categorizations with radically different latent spaces, all humans can distinguish a muffin from a dog, or find the fire hydrants in an image. AI will be inherently better in complex setups, logic, whereas humans perform better in more simple, straightforward setups, with intuitive tasks, producing totally different forms of frustration.

Ideally, the human brain can balance intuition with logic. But when anxiety or fear takes over, the emotional response hijacks our logical thinking. Fear, anger, or any strong emotion overrides our rational processes, causing impulsive decisions and deepening our suffering. None of these emotions are available to an AI. Of course, some humans can also learn to operate primarily relying on logic alone. But that requires a certain amount of training, discipline, and the capacity to update our beliefs. While AI can make limited adjustments, it struggles with this kind of belief revision during inference, though advancements like contextual windows (temporary memory spans that allow models to reason across longer sequences), retrieval-augmented generation (methods that extend reasoning by consulting external information), or reinforcement learning approaches are bridging this gap.

## 4 Updating beliefs brings us to learning

Learning through updates works both for deep learning and human learning. One can say that both humans and AI learn by refining their predictions based on errors. Every “prediction error” or surprise shakes up our understanding of the world, which can lead to happiness and fulfillment but also to disappointment, frustration, or suffering. In a Bayesian sense, our human brain is always recalibrating, and when reality doesn't match our expectations, we feel it as a form of violation or suffering (Fetsch et al., 2013; Padrao et al., 2016).

In neuroscience, such recalibration has been linked to broader theories of consciousness. Tononi's Integrated Information Theory (IIT) proposes that experience arises from how deeply information is integrated within a system (Tononi et al., 2016), while Dehaene's Global Neuronal Workspace (GNW) describes consciousness as the broadcasting of information across distributed neural networks (Dehaene et al., 1998). Both suggest that awareness transforms error into experience: the same prediction gap that merely updates a model in AI can evoke suffering in humans because it is globally integrated into a conscious self-model.

Wondering thoughts can be useful for planning, replaying things, or imagining future scenarios, but they can also make suffering pretty worse than it already is. These thoughts have a way of grabbing you by the neck, locking you into a loop where you're unable to escape that same impending bad outcome. A similar phenomenon appears in reinforcement learning agents that get stuck in repetitive actions. Once caught in a loop, they struggle to break free, much like us, trapped by our fears of the future.

## 5 Illusions to understand AI

Because humans lack complete information, we constantly interpret the world through assumptions and guesses. This limits our accuracy but enables flexible, scalable learning. In contrast, AI systems are bound to structured data, even in reinforcement learning or simulations (Deutsch, 1998). Humans fill in gaps using prior beliefs, shaped by emotion and bias. This inferential strategy mirrors virtual reality illusions, where expectations shape experience. In VR, we experience place illusion (feeling physically present) and plausibility illusion (believing the scene is real) (Slater, 2009). Confidence determines how we accept these illusions. When expectations clash with perception, doubt arises, not just in the illusion, but in our sense of reality itself (Gonzalez-Franco and Lanier, 2017). This is where anxiety emerges: a response to uncertainty in our predictive models.

The therapeutic power of VR reveals something fundamental about the relationship between prediction, illusion, and suffering. When we treat phobias or PTSD through virtual exposure, we are essentially retraining the brain's predictive models in a controlled environment where prediction errors can be managed without overwhelming the patient. The illusion becomes a safe space for recalibration, a way to update beliefs about a threat without actual danger. This works precisely because humans can suffer from prediction errors, and that suffering can be modulated through carefully designed experiences. Perhaps this is why virtual reality has been used to treat hundreds of psychiatric patients with disorders previously considered untreatable without heavy psychotropic drugs (Smith et al., 2022; Freeman et al., 2017). These treatments, which can render patients incompatible with social life and lead to severe adverse reactions, are being gradually replaced or complemented by VR therapies, offering patients new possibilities for relief and a better quality of life.

The parallel to AI hallucinations becomes clearer here. Hallucinations in both AI and humans stem from failures in predictive processing. In schizophrenia, these errors produce false perceptions when the brain's models misalign with sensory input. AI hallucinations are similarly generated when outputs diverge from context or training data. But here lies the critical difference: human hallucinations matter because they threaten the integrity of the self and cause suffering. They require intervention not because they are computationally incorrect, but because they are existentially unbearable. AI hallucinations, by contrast, are merely errors to be corrected, bugs in the system rather than assaults on being. The system does not experience its hallucination as a violation. It simply produces an output that happens to be misaligned with its training distribution.

Prediction errors arise from the complexity of the world, limited data, or flawed inference, whether in human minds or machines. Both systems must navigate uncertainty, but only humans suffer in doing so. Our cognitive models are shaped not only by logic but also by belief, emotion, and meaning. Relinquishing the illusion of control is difficult, yet necessary. AI, in contrast, learns without selfhood and does not experience fear or misalignment.

## 6 One not like the other

Human and artificial intelligences now coexist, drawing on shared resources and influencing one another. Yet we shouldn't forget that in humans, suffering is not only a contemplative state but is often the engine behind physical changes that trigger emotions like empathy, fear, compassion, anger, or love, ultimately leading to action and even hope (Han, 2024). This, however, is not the case for algorithms.

Suffering in humans serves as both warning and catalyst. It signals when something is deeply wrong, not just computationally inefficient, but existentially misaligned. It drives us to change circumstances, seek meaning, connect with others, or fundamentally restructure our goals. An AI receiving negative reward signals will adjust its parameters, but it does not feel the urgency to escape its condition. It has no condition to escape from. The optimization continues regardless of whether the process would be agonizing if experienced consciously.

This distinction matters not only philosophically but practically. If we ever attempt to simulate human suffering in machines (whether to better understand consciousness or to create more “human-like” AI), we must consider the ecological and ethical costs of that endeavor. An infinite AI suffering simulation would incur consumption of finite resources that would translate into actual suffering for humans. Moreover, we would be creating something that optimization alone cannot justify: purposeless pain.

If AI is to help us understand ourselves, it must also learn from the depth of human cognition, where suffering reflects not just error but the absence of meaning. The machine can show us the mechanics of prediction and reward, but only we can experience what it means when those mechanics fail. That gap, between processing and experiencing, may be the most human thing about us.
